# Regulation of gene expression in the genomic context

**DOI:** 10.5936/csbj.201401001

**Published:** 2014-01-29

**Authors:** Taylor J Atkinson, Marc S Halfon

**Affiliations:** a Department of Biochemistry, University at Buffalo-State University of New York, Buffalo, NY 14203, USA; b Department of Biological Sciences, University at Buffalo-State University of New York, Buffalo, NY 14203, USA; c NY State Center of Excellence in Bioinformatics and Life Sciences, Buffalo, NY 14203, USA; d Molecular and Cellular Biology Department and Program in Cancer Genetics, Roswell Park Cancer Institute, Buffalo, NY 14263, USA

## Abstract

Metazoan life is dependent on the proper temporal and spatial control of gene expression within the many cells−essentially all with the identical genome−that make up the organism. While much is understood about how individual gene regulatory elements function, many questions remain about how they interact to maintain correct regulation globally throughout the genome. In this review we summarize the basic features and functions of the crucial regulatory elements promoters, enhancers, and insulators and discuss some of the ways in which proper interactions between these elements is realized. We focus in particular on the role of core promoter sequences and propose explanations for some of the contradictory results seen in experiments aimed at understanding insulator function. We suggest that gene regulation depends on local genomic context and argue that more holistic in vivo investigations that take into account multiple local features will be necessary to understand how genome-wide gene regulation is maintained.

## Introduction

**Figure 1:**
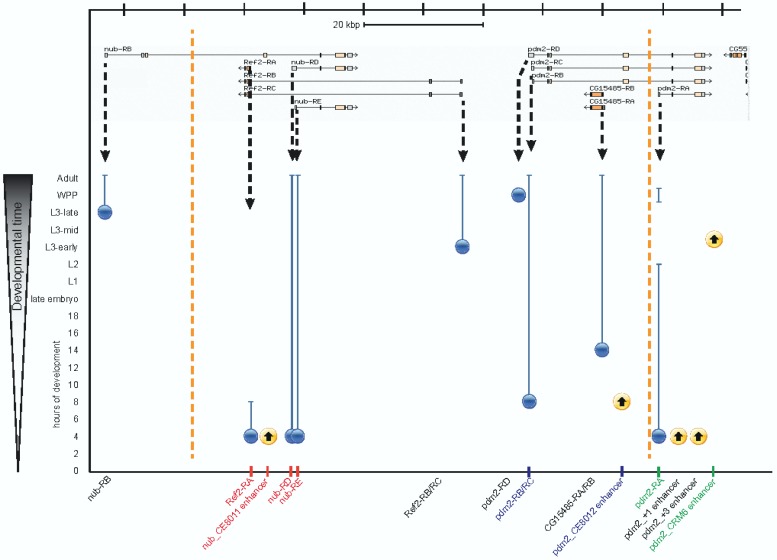
**Genomic region showing promoters, enhancers, and insulators**. Pictured is a 100 kb fragment of the *Drosophila* genome (chr2L:12,593,026..12,693,025), based on the FlyBase v.FB2013_04 genome annotation [102]. Transcripts for the genes *nub, Ref2, pdm2*, and *CG15485* are shown along the top of the figure. Each promoter is highlighted with a vertical black dashed arrow. Insulators are depicted by orange dashed lines and enhancers by yellow circles containing upward-pointing arrows; the arrows connote that while the time when enhancers become active is known, how long they remain active generally has not been described. Enhancer names are drawn from the REDfly database [103]. Developmental time is portrayed vertically on the y-axis; not all stages are shown and the axis is not to scale. Blue circles and lines depict gene expression from each promoter based on RNA-seq data provided as part of the genome annotation. Circles represent the onset of expression and lines continued expression, which sometimes must be inferred as it is not always possible to determine from which promoter the later expression originates. Note that of the seven promoters located between the two insulators, only three appear to be co-regulated, potentially by the *nub_CE8011* enhancer (red text). Promoter *pdm2-RB/RC* may be regulated by the *pdm2_CE8012* enhancer, but the other nearby transcripts are not expressed at the time when this enhancer becomes active (blue text). Interestingly, enhancer *pdm2_CRM6* is active exactly when promoter *pdm2-RA* is inactive, raising the possibility that it engages in insulator bypass to activate one of the other *pdm2* promoters or that its native role is as a negative regulatory element and its classification as an enhancer is due to experimental artifact (green text).

Maintaining proper control of gene expression is fundamental for all organisms. Although much is known about how individual metazoan genes are regulated, how correct patterns of gene activation are maintained genome-wide is not well understood. Every gene lies adjacent to another gene, and many genes have multiple differentially regulated transcripts. Genes can be nested inside other genes or overlap one another on opposite strands of the DNA. Within the nucleus, chromatin is arranged in a three-dimensional fashion such that genes that are far apart on the chromosome, or are on different chromosomes, become closely juxtaposed. Given such complexity in genomic organization, it is a wonder that gene expression can be correctly sorted out: when regulatory elements are able to act over large distances and ignore intervening elements, how is one regulatory element able to target a specific gene while at the same time bypassing other nearby promoters? We consider here some answers to this question. Our focus is not on broad epigenetic mechanisms such as heterochromatic silencing and Polycomb-mediated repression of large chromatin domains (reviewed by [1]) but rather on local-scale events such as the differential expression of several genes lying in an apparently similar chromatin state or physical region (Fig. 1). We begin with a brief review of the main regulatory elements that influence gene expression and genomic organization—promoters, enhancers, and insulators—followed by a discussion of possible mechanisms for ensuring faithful gene regulation. We highlight the often overlooked role of core promoter sequences in mediating specific enhancer-promoter interactions and describe some of the challenges of trying to understand genome-wide events using approaches centered on single genes or regulatory elements. We suggest that a more holistic view of regulation, taking into account the full set of local genomic features, will be needed to fully understand how gene expression throughout the genome is properly maintained.

## Promoters

Required for the transcription of eukaryotic RNA polymerase II-transcribed genes is the core promoter, typically defined as consisting of the DNA approximately 35-40 bp upstream and downstream of the transcription start site (TSS) [2]. This is at least in part a functional definition in that this region is usually sufficient to mediate gene expression in a reporter gene assay. The core promoter contains sequence elements, referred to as “core promoter motifs,” which interact with the basal transcription machinery, including RNA polymerase II and the TFIID complex (reviewed by [3, 4]). Although a number of prevalent core promoter motifs have been defined, there are no universal motifs common to all promoters, and the majority of promoters do not contain any currently-identified motifs [5]. Arguably the best-known core promoter motif is the TATA box, which may be present in from 5-20% of mammalian promoters [6, 7] and which binds the TFIID component TATA-box binding protein (TBP). Additional motifs include the TFIIB recognition BRE elements, the Inr motif, and the DPE (downstream promoter element) motif. FitzGerald et al. [8] defined 15 core promoter motifs in *Drosophila* and eight in human, with only TATA, Inr, and DPE clearly present in both species. However, a similar study by Gershenzon et al. [9] also observed commonality of BRE and DCE between the two species. These differences help to underscore the difficulty of motif-based analyses, in which motif quality, motif degeneracy, choice of statistical cutoff scores, and size and composition of the sequence search space all impact the end result.

Large-scale mapping of TSSs has revealed different classes of promoters based on TSS distribution [10–12]. “Single” or “narrow” peak promoters are distinguished by a tight cluster of TSSs spanning only one or several basepairs, whereas for “broad, ” “weak,” or “wide” peak promoters the TSSs are distributed over a wide range, up to 100 bp. Intermediate classes such as “broad with peak” or “multimodal” have also been observed. TSS clusters are considered as distinct from alternative promoters, which show a clear spatial separation from one another, have their own core promoter regions, and give rise to distinct, individually annotated transcripts of the same gene. It should be noted that canonical core promoters are only well-defined for narrow-peak promoters, and it is not clear exactly how the core promoter region for a broad-peak promoter should be defined. Part of the problem is that narrow peak promoters are generally the ones associated with position-specific core promoter motifs such as the TATA box, Inr, and DPE, whereas broad peak promoters are more likely to have location-independent motifs and (in mammals) CpG islands [10–13]. This lack of position-specific motifs makes it difficult to define the core promoter with any precision for the broad-peak promoters absent extensive experimental determination, which has not so far been undertaken. Acetylation of histone 3 lysine 9 has also been shown to distribute differently among the different promoter shape classes [14]. These sequence and biochemical differences appear to be related to functional differences: housekeeping genes tend to have broad peak promoters, whereas tissue-specific gene promoters are more frequently narrow peak.

In addition to the core promoter, studies have suggested a contributory role in proper gene regulation for the extended promoter region of up to approximately 350 bp upstream of the TSS (e.g. [6]). Although it is possible that this simply reflects the activity of the closest-lying distal *cis*-regulatory modules (enhancers), differences in nucleotide composition are also observed in this region relative to more upstream sequences in both flies and humans, suggesting that the proximal promoter does indeed represent a distinct functional region [5].

## Enhancers

Although a promoter is absolutely required for gene transcription, a significant part of metazoan transcriptional regulation occurs via the action of distal *cis*-regulatory modules. The best studied of these are transcriptional enhancers, distal non-coding sequences that positively regulate transcription. As originally defined, enhancers act without regard to orientation, distance, or placement (5’/3’) relative to the transcribed gene [15]. In practice, however, the term ‘enhancer’ is often used loosely to mean any positive-acting regulatory element, without explicit confirmation of distance or orientation independence. Enhancers are frequently modular; a gene with a complex expression pattern may have a large number of enhancers, each up to a few hundred basepairs in length and each responsible for a discrete spatiotemporal aspect of the gene's expression. Many genes also possess seemingly redundant, or functionally highly similar, enhancers, which in at least some cases have been shown to increase fidelity or robustness of gene expression [16–18]. Enhancers serve as a platform for the assembly of transcription factors, including activators, repressors, and chromatin modifying enzymes. The dominant model of enhancer function is that enhancers act by means of DNA looping, forming contacts with the promoter in order to either stabilize RNA polymerase binding or mediate release of stalled polymerase. Enhancers have been the subject of several recent comprehensive review articles, and the reader is referred to these for further details [19–23].

There is growing evidence that enhancers are marked in the genome by specific sets of histone modifications, in particular monomethylation of histone 3 lysine 4 (H3K4me1) and acetylation of histone 3 lysine 27 (H3K27ac) [24]. These observations have been used to undertake widespread enhancer discovery in human cell lines and provide what is likely a lower-bound estimate of over 400,000 enhancers spanning over 10% of the genome [25]. Histone modification patterns have also been used to make inferences about the activity state of enhancers, including “active” and “poised” elements (e.g. [26, 27]). However, definitive interpretation of the relationship between specific chromatin modifications and enhancer activity status should be treated with caution, as many of the current results are obtained from large-scale genomic analysis of cultured cells with relatively little in vivo validation. Indeed, many of the “enhancer” sequences are not verified but rather inferred from histone modification or chromatin accessibility, leading to a certain amount of circularity in the data analysis. For example, enhancers may be predicted based on one set of histone modifications, but then that same predicted set may be used to evaluate if an additional feature is also associated with enhancers, without there being a true validation step in between. In one case where a set of enhancers with well-described spatiotemporal activity was examined using cells isolated from intact animals, a range of histone modifications was found to be associated with both active and inactive enhancers, and some active enhancers did not seem to have any of the described chromatin states [28]. Thus, the true range of chromatin modifications associated with enhancers is likely to be both complex and dynamic, and significant further investigation is needed.

Genome-scale transcriptional profiling has made it apparent that enhancers are often if not always transcribed into RNA. This coupling was initially remarked on by Li et al. [29] for *Drosophila* enhancers, and subsequently shown more directly in a number of mammalian systems [30–32]. The prevalence of non-coding transcription in the genome makes it difficult to determine if these “eRNAs,” as they have become known, represent a single class of transcripts or a combination of different transcripts arising from different mechanisms, and much remains to be understood about enhancer-related transcription. However, a consensus has begun to develop around eRNAs as bidirectional, non-poyladenylated RNAs [reviewed by 33]. The functional importance of eRNAs also remains unresolved. Although several studies have reported a role for the transcripts themselves in inducing gene expression [e.g. 34,35,36], other studies suggest that it is the act of transcription which is important for enhancer activity, with the transcripts themselves merely a byproduct [37]. Still other studies find that neither transcription nor transcript is required, and that eRNAs merely represent transcriptional “noise” [38]. It may well prove that all of these conclusions are correct, with multiple different transcriptional mechanisms contributing to the observed widespread enhancer transcription.

## Insulators

A third critical component contributing to global fidelity of gene expression is the insulator. Originally defined in *Drosophila*, and still best understood in that organism, insulators were so named due to their ability to “insulate” genes from position effects in transgenic assays. Historically, two major roles have been ascribed to insulator elements: the ability to serve as boundary elements preventing the spread of heterochromatin, and the ability to prevent enhancer activity when interposed between an enhancer and promoter. It was subsequently demonstrated that insulator elements act to promote DNA looping, suggesting that their mechanism of action might be to sequester regions of chromatin into discrete domains. In recent years, bolstered by large-scale chromatin conformation assays that have allowed for genome-wide identification of chromatin architecture (reviewed by [39]), this has come to be viewed as the primary function of insulator elements and, in fact, the more traditional boundary and enhancer-blocking roles have been called into question (see below). Detailed current views of insulator function can be found in any of a number of several excellent recent reviews [40–43].

A large cohort of DNA-binding proteins have been associated with insulator activity in *Drosophila*, including Su(Hw), ZW5, GAF, BEAF-32, CP190, Mod(mdg4), and CTCF [44–50]. Several studies have demonstrated that these proteins often bind in concert, implying cooperativity, although there is conflicting evidence as to which are the predominant combinations and what effect this has on function. Negre et al. [51] described two insulator classes based on ChIP-seq experiments, the first consisting of BEAF-32, CP190, and CTCF binding sites and the second of Su(Hw)-associated sites; GAF showed limited clustering with the other factors. However, Schwartz et al. [52], based on similar data, define 16 binding classes. What if any functional distinctions exist between these classes has not been established. The differences lie largely in different treatments of binding strength and interpretations of combinatorial binding. The latter results suggest the possibility of a diversity of insulator roles and functions based on different combinations of insulator binding proteins. Until some of these questions are resolved, care should be taken in making functional inferences based on currently annotated *Drosophila* insulator elements, which do not take differences in insulator protein binding into account.

In contrast to the large variety of insulator proteins identified in *Drosophila*, insulator function in mammals appears to be primarily carried out by CTCF. CTCF, a large and ubiquitously-expressed zinc finger protein [53], has been shown to be involved in the formation of chromatin loops and chromatin architecture generally (reviewed by [43, 54]). Global mapping of CTCF-mediated interactions conducted in mouse embryonic stem cells revealed extensive chromatin looping at multiple scales and suggests that CTCF plays an important role not just in the classical insulator sense of preventing enhancer-promoter interactions but also in facilitating such interactions to promote gene expression [55]. CTCF associates with cohesin, and the cohesin complex may be required for chromatin loop formation, and hence insulator function, mediated by CTCF (reviewed by [56]). In *Drosophila*, CP190 is frequently associated with CTCF at insulators and may play a role analogous to that of cohesin in mammalian cells [43, 57, 58]. Also able to function as mammalian insulators are binding sites for the RNA polymerase III transcription factor TFIIIC, both at clustered tRNA genes and at individual TFIIIC bound sites, including those located in short interspersed nuclear elements (SINEs)^59^. Like CTCF insulators, TFIIIC insulators are found in complex with cohesin, suggesting an overall similar mechanism for insulator activity [60]. TFIIIC may represent the most ancient and conserved insulator function, acting as such at least as far back as yeast [61].

## Do insulators have primary responsibility for maintaining appropriate gene expression?

A growing amount of evidence has begun to raise questions as to the centrality of the traditionally-conceived role of insulators as both preventers of heterochromatic spreading and enhancer-blockers. Despite a provocative correlation of CTCF and other insulator proteins with the borders of epigenetically distinct chromatin domains, mutation or RNAi-based depletion of these factors has little effect on chromatin state boundaries, although a small number of loci do show spreading of the repressive chromatin mark H3K27me3 [52, 62, 63]. Thus, while insulators may serve a chromatin boundary function in certain instances, this does not appear to be a primary role of these elements. Similarly, RNAi knockdown of insulator proteins fails to reveal significant global changes in gene expression, a difficult result to rationalize if insulator-mediated enhancer blocking provided a major mechanism for preventing inappropriate activation of genes by nearby enhancers [52]. In one of the few direct in vivo experiments that have been performed, Soshnev et al. [64] created a genomic deletion of a sequence that had been shown in a transgene assay to function as a Su(Hw)-dependent insulator. Surprisingly, deletion of the sequence failed to affect expression of adjacent genes. Moreover, only a small fraction of insulators defined via insulator protein binding in ChIP-seq assays have been found to display consistent enhancer-blocking function in standard transgene insulator activity assays [52, 65].

On the other hand, the clear ability of some insulators to function as enhancer-blockers in transgenic assays coupled with striking albeit circumstantial data based on insulator positioning leave the possibility of a wide-spread role of insulators as enhancer blockers an enticing one. For instance, Negre et al. [51] show under-representation of insulators between enhancers and their target promoters and enrichment of insulators between enhancers and non-target promoters. They additionally demonstrate that insulators are more prevalent between differentially expressed promoter pairs than between similarly expressed promoter pairs, as would be expected if insulators were playing a role in enhancer blocking. Yang et al. [66] examined adjacent genes with a “head to head” configuration and found enrichment for BEAF-32 binding only in those pairs that were not co-expressed. Although they did not test these sequences for enhancer blocking activity or monitor changes of gene expression following BEAF-32 depletion, the strong correlation is difficult to discount.

One complicating factor is that insulator activity may be a regulated dynamic function rather than a fixed property. This has been observed in *Drosophila*, where the formation of insulator bodies, nuclear sites where multiple individual insulator sites are seen to aggregate, is disrupted following heat shock as a result of relocalization of CP190 away from insulator sites [67, 68]. In a similar vein, hormone stimulation results in changes of insulator protein binding at a subset of sites, with concomitant subtle changes in the expression of surrounding genes [68]. The mechanisms responsible for the observed protein relocalization are currently unknown. CTCF function in vertebrates can be modulated by co-factor binding [69], whereas CTCF binding is influenced by DNA methylation [70, 71] and by transcription through CTCF binding sites [72]. As a result, transgenic assays for insulator function may fail due to missing factors or non-permissive conditions, leading to an erroneous conclusion that a tested site is unable to mediate enhancer blocking activity. Additional assay-specific complications are discussed below (“genomic context”).

## Enhancer-Promoter Specificity

Even under a best-case assumption about the enhancer-blocking ability of most insulator elements, we are far from explaining just how appropriate gene expression is maintained throughout the genome. For example, making the extreme assumption that all of the annotated insulators in the *Drosophila* genome [51] are accurately identified and contain enhancer blocking activity, we still find that over half of all promoter pairs lying between two insulators have completely uncorrelated activity (MSH, unpublished data; see example in Fig. 1). Therefore, either the majority of enhancer blocking insulators have yet to be identified, or additional mechanisms, other than chromatin insulators, have primary responsibility for restricting enhancer activity to the proper promoters.

An aspect of gene regulation that is likely to be important, although frequently overlooked, is the role played by specific compatible/incompatible enhancer-promoter pairings, that is, enhancers that are capable of activating only certain promoters. Although the existence of such specific enhancer-promoter interactions is well known [73–78], this has been considered to be an exception to, rather than a norm of, the mechanisms used to restrict enhancer activity [79]. However, there is reason to believe that enhancer-promoter specificity may be significantly more prevalent than commonly assumed. In addition to the widely uncorrelated promoter activity described above, a genome-wide survey of core promoter motifs in *Drosophila* showed that the promoter sequences of neighboring genes are more similar than expected by chance (even after accounting for gene duplication), with a strong correlation between core promoter motif similarity and both strength and pattern of gene expression [5]. These findings suggest that common enhancers might be regulating the adjacent genes with similar promoters, but not those with dissimilar promoters. Gehrig et al. [80] tested ten different enhancers in reporter gene assays in zebrafish, using 18 different promoters with each. For at least eight of the enhancers they were able to identify one or more incompatible promoters. Similarly, in a test of 18 *Drosophila* enhancer-trap loci, Butler and Kadonaga [73] found that four (22%) showed specificity for one of two tested core promoters.

There is clear evidence that core promoter motifs play a key role in determining enhancer-promoter specificity, in particular with respect to the well-described *Drosophila* TATA and DPE motifs, which for at least some tested enhancers signify a mutually exclusive set of compatible promoters [73, 74, 77, 78]. Less is known about what mediates specificity from the perspective of the enhancer. The transcription factor Caudal has been shown in *Drosophila* to preferentially activate promoters with the DPE motif as compared to the TATA motif and especially the combination of TATA and BRE^u^ motifs [81]. However, the molecular interactions underlying this preference remain uncharacterized. In general, the mechanisms mediating enhancer-promoter specificity remain poorly understood.

## Genomic context

Enhancer promiscuity has been believed to be the rule because in reporter gene assays, most enhancers are able to activate heterologous promoters. However, in truth, few enhancers are tested with a variety of different promoters, and the evidence for heterologous promoter activation is to some extent circular: sequences that fail to activate gene expression in a reporter gene assay are typically labeled as “non regulatory” precisely due to their failure to drive expression from a heterologous promoter. Even granting a general lack of promoter specificity in reporter gene assays, however, it is distinctly possible that such assays do not realistically reflect the situation confronted by an enhancer in its true genomic context.

### Distance

At least two major differences come into play when comparing typical reporter gene assays to actual genomic scenarios. One, enhancers in reporter constructs are usually placed in proximity to the promoter, rarely more than several hundred basepairs and often less than 100 basepairs away. Although by formal definition “enhancers” are distance-independent [15], in practice distance effects, ranging from attenuation to complete elimination of enhancer activity as distance increases, have frequently been observed when assayed. The mechanisms responsible for distance-dependence are not well understood, but at least three classes of elements have been described in *Drosophila*. One class comprises the “promoter tethering elements” [78, 82–85]. These elements lie in proximity to the promoter and are responsible for mediating interaction with appropriate distal enhancers. A tethering element in the *engrailed* *(en)* locus contains Polycomb response elements (PREs), and the presence of PREs is potentially a contributing factor in the ability of *en* enhancers to act at a distance [78]. Of three assayed genes whose promoters contain both Inr and DPE motifs, both *en* and *invected* were activated by the *en* enhancers, and both have closely linked PREs. In contrast, *sprt*, which has a similar promoter but no PRE, failed to respond. The requirement for the PRE suggests that Polycomb-group proteins may be involved in promoter tethering, although whether this is through the chromatin-modifying activity of the Polycomb complex or a different mechanism is currently unknown. In the *white* locus, binding of Zeste to a promoter-proximal region is required for long-range interactions with the *white* eye enhancer, but is dispensable when the enhancer is moved close to the promoter [86]. Interestingly, this same interaction displays insulator bypass, i.e., it allows activation of the promoter despite an intervening insulator. How common tethering elements may turn out to be, and whether the presence of PREs and/or Zeste binding sites are defining features of this class of elements, remains to be determined.

The second class consists of the “promoter targeting sequences” (PTS) [87]. The two known PTS elements both lie within the *Drosophila Abdominal-B* locus and have two activities: they allow for long-distance enhancer-promoter interaction and for restricting this interaction to a single promoter. The PTS, similar to the Zeste-binding promoter tethering element described above, are able to mediate insulator bypass; in fact, insulator presence may be a requirement for initial PTS activity [88, 89]. Interestingly, presence of an insulator is not necessary for maintenance of PTS activity, suggesting that the PTS may function via epigenetic modification of local chromatin and/or formation of a stable chromatin loop [88]. However, these mechanisms have not yet been tested. PTS elements differ from promoter tethering elements mainly in location: the latter are promoter-proximal whereas the former are more enhancer-proximal. It is not yet known whether both utilize a similar mechanism of action or if they represent truly different classes of elements.

The third class, for which there is currently only a single example, is the “remote control element” (RCE) found in the *Drosophila shaven (Pax-2) “sparkling”* enhancer [90]. The RCE is completely dispensable for enhancer activity when near (∼ 100 bp) the promoter, but essential for activation at a distance (> 800 bp). As few enhancers have been subject to the intensive mutagenesis-based analysis carried out for the *sparkling* enhancer, no less tested at multiple distances from the promoter, it remains to be seen whether the RCE represents a rare or common mechanism for insuring enhancer-promoter specificity.

### Promoter competition

The second, and perhaps even more relevant, difference between reporter assay and genomic context is the presence of additional flanking promoters, which may compete with one another for activation by an enhancer. In human erythrocytes, the *alpha-globin MCS-Rs* enhancer regulates the *NME4* gene, located 300 kb distal, in addition to the more closely located *alpha-globin* genes, as evidenced both by gene expression and physical interaction between the enhancer and the *NME4* promoter [91]. If the *alpha-globin* genes are deleted, expression of *NME4* is upregulated and the *MCS-Rs/NME4* interaction increases in strength. These results suggest that competition between the more proximal *alpha-globin* promoters and the distal *NME4* promoter favors interaction of the enhancer with the former. When the competing *alpha-globin* promoters are no longer present, the enhancer is released for increased interaction with (and hence activation of) the *NME4* promoter. Other promoters lying between *alpha-globin* and *NME4* are unaffected.

**Figure 2:**
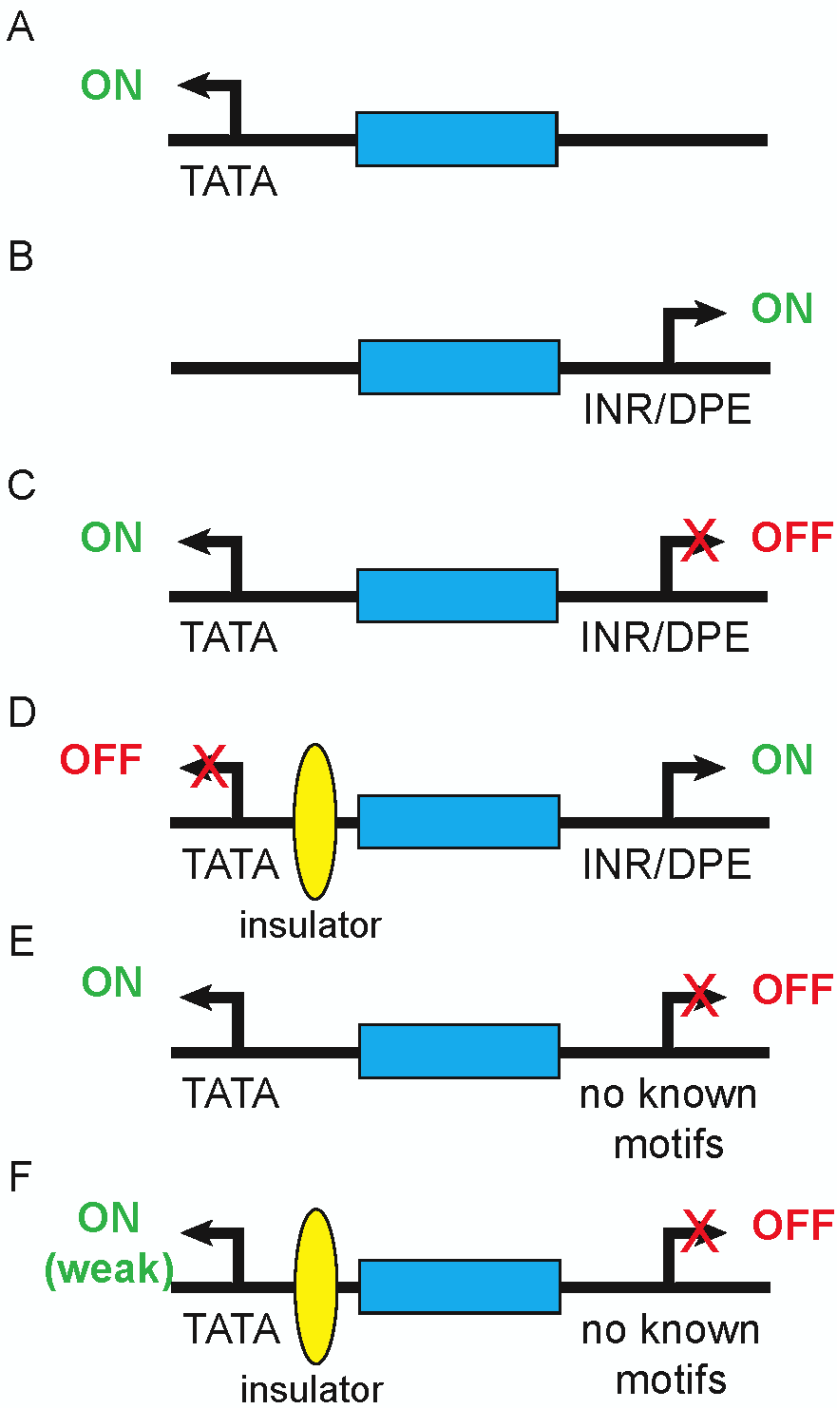
**Promoter competition experiments** (adapted from [66, 81]). Arrows represent promoters, with key core promoter motifs listed below. Blue boxes represent enhancers, ovals insulators. (A) The enhancer is able to activate a TATA-containing promoter as well as (B) an INR/DPE-containing promoter, when either is the only promoter present in proximity. (C) When both promoters are placed equidistant from the enhancer, only the TATA promoter is activated. (D) Placement of an insulator between the enhancer and TATA promoter blocks activation of this promoter and restores the ability of the enhancer to activate the INR/DPE promoter. (E) A different promoter, which does not contain any known core promoter motifs, does not compete effectively with the TATA promoter and (F) allows it to be activated even in the presence of an intervening insulator. The strength of the activation (degree of insulator bypass) may depend on how strongly the other promoter is able to compete for the enhancer.

Working in *Drosophila*, Ohtsuki et al. [77] demonstrated that an enhancer which in isolation is compatible with either of two different promoters might interact exclusively with just one when offered a choice between them (Fig. 2A–C). Not all promoters provide competition, and preference for one promoter over another is determined at least in part by core promoter motifs. Lee and Wu [74] used an elegant transvection assay to arrive at similar conclusions. Thus, even though an enhancer may appear to function with a given promoter in isolation in a reporter assay, in its genomic context the same enhancer might be prevented from activating that promoter due to the neighboring presence of a more preferred partner. Considering jointly the potential effects of both distance between an enhancer and promoter and competition from other nearby promoters suggests a complex range of parameters that factor into promoter choice, and which could lead to distinct effects in a native genomic context not observed in reporter assays utilizing a single heterologous promoter.

### Promoters, enhancers, and insulators together in context

Promoter competition and distance have also been shown to influence insulator activity. Building on the assay developed by Ohtsuki et al. [77], Cai et al. [92] demonstrated that a Su(Hw) insulator displayed effective enhancer-blocking activity in the presence of a strong competitor promoter, i.e., a promoter with clear preference for the enhancer (Fig. 2C, D). However, the identical insulator had much weaker enhancer-blocking function when the challenging promoters were non-competitive: an insulator interposed between an enhancer and its preferred promoter had reduced or even no effect when the second flanking promoter was not compatible with the enhancer (Fig. 2E, F). Indeed, insulator-mediated enhancer blocking activity ranged from strong to moderate to none depending on the degree of similarity between the promoters. This implies that not only may promoters with sufficiently different sequences not require an intervening insulator to keep from being activated by a common enhancer, but that an insulator, even if present in such a situation, may have limited or even no activity. Similarities between insulators and promoters have been remarked upon—promoters can mediate both chromatin-barrier and enhancer-blocking functions, and both elements are involved in the formation of chromatin loops—and selectivity in enhancer-promoter interactions may depend on local chromatin conformation and relative affinity for loop formation among all three of enhancers, promoters, and insulators [93]. Consistent with this, Maksimenko et al. [94] found that the arrangement of multiple nearby insulator sites, as well as the respective distances between enhancers, insulators, and promoters, could significantly affect the degree of enhancer blocking conferred by the insulators. These findings suggest possible explanations for some of the contradictory results discussed above with respect to putative insulators not appearing to function as such in transgene assays: some of the tested insulator sequences may only function in the presence of a sufficiently competitive second promoter, or may be affected by the relative spacing of the various regulatory components combined in the assay. Considering that many genes have multiple alternative promoters which (compared to promoters of different genes) are spaced relatively close together, the potential impact of promoter competition or of promoters embodying insulator-like enhancer blocking functions is considerable. Determining the extent to which this is so will need to await the development of more complex insulator assays that take into account promoter strength, promoter competition, and enhancer-promoter distance.

## Summary and Outlook

Transcriptional regulation takes place within a complex genomic milieu in which enhancers, promoters, and insulators are closely connected both along the one-dimensional linear chromosome and within the three-dimensional nuclear chromatin environment. To date, our understanding of regulatory events has been limited by the constraints of the assays available for their exploration. Cell culture-based studies have the advantage of lower noise in genome-scale assays, but may not always faithfully recapitulate in vivo conditions. In vivo studies, on the other hand, usually entail simultaneous examination of multiple cell types. These assays therefore suffer from reduced sensitivity to the events occurring in any one cell type and enable only an averaged picture of what might in fact be discrete interactions taking place in different cells. Meanwhile, in either system, typical reporter gene assays using closely-linked enhancers and a single promoter may not sufficiently recapitulate the genomic environment of promoter competition and enhancer action-at-a-distance to provide a realistic picture of how regulatory elements are functioning in their native context. Fortunately, there have been substantial recent developments in methodology for single-cell assays [95, 96] and genome engineering [97]. Single-cell assays offer the possibility of determining transcriptional and epigenetic profiles for specific cell types isolated from primary tissue rather than cell lines, ultimately allowing for investigation of a wider range of cell types as well as developmental time-series analysis and other high-resolution spatial and temporal analyses not currently possible. Genome engineering methods such as transcription activator-like effector nucleases (TALENS) and the RNA-guided CRISPR/Cas9 system enable precise deletion or mutagenesis of regulatory sequences in a wide selection of model organisms. Moreover, transcription activator-like effectors (TALEs) have been used to great effect to target activators or repressors to enhancer sequences to modulate their function [98] as well as to induce specific epigenetic modifications in a sequence-specific manner [99–101]. In concert, these methods hold great promise for enabling a new generation of detailed and holistic in vivo investigations that may resolve some of the contradictions in the current data and shed new light on how proper gene expression is maintained within the genomic context.
